# Reproducibility of isokinetic strength assessment of knee muscle actions in adult athletes: Torques and antagonist-agonist ratios derived at the same angle position

**DOI:** 10.1371/journal.pone.0202261

**Published:** 2018-08-15

**Authors:** João P. Duarte, João Valente-dos-Santos, Manuel J. Coelho-e-Silva, Pedro Couto, Daniela Costa, Diogo Martinho, André Seabra, Edilson S. Cyrino, Jorge Conde, Joana Rosado, Rui S. Gonçalves

**Affiliations:** 1 Research Unity for Sport and Physical Activity (CIDAF, UID/DTP/04213/2016), University of Coimbra, Coimbra, Portugal; 2 Faculty of Sport Sciences and Physical Education, University of Coimbra, Coimbra, Portugal; 3 Portuguese Foundation for Science and Technology (SFRH/BD/101083/2014), Lisbon, Portugal; 4 Portuguese Foundation for Science and Technology (SFRH/BPD/100470/2014), Lisbon, Portugal; 5 Institute for Biomedical Imaging and Life Sciences (IBILI), Faculty of Medicine, University of Coimbra, Coimbra, Portugal; 6 Faculty of Physical Education and Sport, Lusófona University of Humanities and Technologies, Lisbon, Portugal; 7 Portuguese Foundation for Science and Technology (SFRH/BD/121441/2016), Lisbon, Portugal; 8 Research Centre in Physical Activity, Health and Leisure (CIAFEL), Faculty of Sport, University of Porto, Porto, Portugal; 9 Portugal Football School, Portuguese Football Federation, Lisbon, Portugal; 10 Metabolism, Nutrition, and Exercise Laboratory (GEPEMENE), State University of Londrina (UEL), Londrina, Brazil; 11 School of Health and Technology, Polytechnic Institute of Coimbra, Coimbra, Portugal; 12 Centre for Health Studies and Research, University of Coimbra, Coimbra, Portugal; University of Debrecen, HUNGARY

## Abstract

The current study aimed to examine the reliability of the conventional and functional ratios derived from peak torques (PTs) and those obtained from the combination of knee flexors torque at the angle of knee extensors PT. Twenty-six male athletes (mean of 24.0±0.7 years) from different sports completed a test-to-test variation in isokinetic strength (Biodex, System 3) within a period of one week. Anthropometry and body composition assessed by Dual Energy X-ray Absorptiometry were also measured. The proposed isokinetic strength ratio measurements appeared to be highly reliable: conventional ratio at PT angle (intra-class correlation, ICC = 0.98; 95% confidence interval; 95%CI: 0.95 to 0.99); functional extension ratio at PT angle (ICC = 0.98; 95%CI: 0.96 to 0.99); and, functional flexion ratio at PT angle (ICC = 0.95; 95%CI: 0.89 to 0.98). Technical error of measurement (TEM) and associated percentage of the coefficient of variation (%CV) were as follows: conventional ratio at PT angle (TEM = 0.02; %CV = 4.1); functional extension ratio at PT angle (TEM = 0.02; %CV = 3.8); and, functional flexion ratio at PT angle (TEM = 0.03; %CV = 3.6). The current study demonstrated that the traditional and new obtained simple and combined isokinetic indicators seem highly reliable to assess muscle strength and function in adult male athletes. A single testing session seems to be sufficiently to obtain these isokinetic strength indicators.

## Introduction

The terms muscle strength and muscle power are erroneously used as synonymous in many professional contexts. In the present study, the term ‘muscular strength’ refers to maximal muscular force in a single voluntary contraction [1]. In the meantime, torque corresponds to the ability of a force to cause rotation on a lever. The relationship between the torque exerted by a muscle group and the range of motion of the joint is determined by mechanical characteristics of the anatomical lever system. In many muscle actions, such as knee extension and knee flexion, the mechanical disadvantage of the muscles occurs at the extremes of the range of motion. Isokinetic refers to dynamic muscular contraction characterized by a constant angular velocity of the movement [2, 3]. The angular velocity is kept constant by the dynamometer that adjusts the resistance applied to the muscles through the range of motion (i.e. the load applied to the muscle is increased at the point of highest mechanical advantage of the muscle and, correspondingly, the load is decreased at the extremes of the range of motion).

Several parameters are cited in the literature as a measure of isokinetic strength. Peak torque (PT) is consistently favored as the most prominent information retained for analysis [4, 5] and the angle of its occurrence is often reported [6–8]. Meanwhile, mean torque corresponds to mean values of the moment of force during a particular range of motion. Additionally, and regarding the knee joint, the strength ratios tend to be calculated by dividing the maximal knee flexors (KF) moment by the maximal knee extensors (KE) moment measured at identical angular velocity and concentric (cc) or eccentric (ecc) contraction actions. In other words, the following ratio is usually termed as conventional ratio: KFcc/KEcc [9]. Two other ratios were also suggested to examine the antagonist-agonist strength relationship for knee extension and knee flexion actions. They were termed as functional extension ratio and functional flexion ratio, respectively KFecc/KEcc and KFcc/KEecc [9]. Active quadriceps muscle contraction may create significant anterior tibial translation or shear and it may also produce substantial internal rotation of the tibia relative to the femur [10, 11]. The amount of co-activation of the hamstring muscles was suggested [12] to be crucial for ligamentous constraints, particularly the anterior cruciate ligament.

The conventional and functional ratios are being derived from the respective PTs that occurred at different angles for KE and KF. However, the antagonist-agonist strength relationship should be interpreted at the specific angle, particularly, at the angle of the agonist PT. Because of these data quality properties are of utmost importance in the evaluation of athletes, the purpose of the current study was to examine the reliability of the conventional and functional ratios derived from PTs and those obtained from the combination of KF torque at the angle of KE PT (the denominator in all composite variables).

## Materials and methods

### Research design and procedures

The present study required repeated measurements with one week apart. The local Ethics Committee (CE/FCDEF-UC/00182016) previously approved the research project. Standards for research in sports medicine were followed taking into account the Declaration of Helsinki. Participants were informed about the objectives of the study, protocols and risks related to data collection. All provided written informed consent, which was approved by the ethics committee before the beginning of the study. Participants were informed that they could withdraw from the experiment at any time. Verbal consent was provided during the second test session to ensure voluntary participation. All measurements were performed at the Laboratory of Biokinetics at the Coimbra University Stadium and participants were instructed to avoid food ingestion for at least three hours before testing and not to drink caffeine during the day. All assessments were performed at the same time of the day (10:00–12:00 a.m.).

### Participants

All participants were recruited in the Coimbra University Stadium according to the following inclusion criteria: males, aged >17 years and <36 years; 2+ years of training experience in competitive sports; none had any history of severe time-loss injury in the previous two months; none were taking any medication or supplements known to affect performance. Chronological age was determined to the nearest 0.01 year by subtracting birth date from date of first testing measurement. Training experience was obtained by questionnaire. The sample was composed of 26 adults aged 18.6–33.9 (mean of 24.0±0.7 years) with different training history [soccer (n = 12), combat (n = 4), swimming (n = 3), rowing (n = 2), track and field (n = 1), tennis (n = 1), volleyball (n = 1), cycling (n = 1), and roller hockey (n = 1)]. Sample size was similar to previous studies aimed to examine the reproducibility of isokinetic and isometric knee extensor and flexor muscle strength [13].

### Anthropometry

All measurements were obtained by a single experienced observer using standard procedures [14]. Body mass was assessed using a *SECA* portable scale (model 770, Hanover, MD, USA) with an accuracy of 0.1 kg. Stature was measured using a *stadiometer Harpenden* (model 98.603, Holtain Ltd, Crosswell, UK) to the nearest 0.1cm. A portable table (Harpenden, model 98.607, Holtain Ltd, Crosswell, UK) was used to measure sitting height to the nearest 0.1 cm. Leg length was estimated as stature minus sitting height.

### Body composition

Dual Energy X-ray Absorptiometry was used to assess body composition (Lunar DPX-MD+, Software: enCORE version 4.00.145, GE Lunar Corporation, Madison, WI, USA). All athletes were assessed in the supine position during a visit to a certified laboratory always performed during the mornings. An experienced technician supervised all assessments. The technology provides information regarding bone mineral content, bone mineral density, fat tissue and lean soft tissue. Extracted data for this study included information reporting whole body and lower limbs (dominant lower limb).

### Isokinetic dynamometer

The protocol starts with a 5-min warm-up in a cycle ergometer (814E Monark, Varberg, Sweden) using a braking force corresponding to 2% of the body mass [15]. The cadence was kept between 50 and 60 rpm. Afterwards, static stretching was done for the quadriceps, hamstrings and adductors (20 seconds each position). Biodex System 3 dynamometer (Shirley, NY, USA) was used to assess the isokinetic strength of KE and of the KF from the dominant lower limb at an angular velocity of 60°·s^-1^. This isokinetic dynamometer is a reliable and valid instrument [16]. Participants were seated in the dynamometer according to manufacture guidelines, that is, adopting a standardized position of 85° hip flexion from the anatomical position. The lever arm was aligned with the lateral epicondyle of the knee. The trunk, dominant thigh and leg (slightly above the medial malleolus) were stabilized with belts. Range of motion was defined for 85° degrees (knee flexion 5° to 90°) as follows: athletes were asked to perform a voluntary maximal knee extension and the 0° was settled; afterwards, the initial 5 degrees of the flexion were completed and the dynamometer blocked. The reduction of the initial angles of the flexion was done to allow the athlete to exert at least 10% of the assigned torque limit. Prior to each test, individual calibration was completed for gravity correction [17]. This correction was determined at the position of 30° of knee flexion. During the test, participants were instructed to keep the arms crossed with the hands on the opposite shoulder [18]. A specific 3-repetition trial was performed prior to each isokinetic test [19], to reduce the effect termed “familiarization”. Finally, the test was completed with real-time visual feedback given by the screen of the dynamometer [18]. Reciprocal cc and ecc muscular actions were tested considering 5 repetitions for knee flexion and knee extension at 60°·s^-1^ (1.05 rad·s^-1^). A 60-second interval was settled between the 3-repetition familiarization and the test [20]. The sequence was: 3-repetition knee extension cc combined with knee flexion cc; 60-second interval; 5-repetition knee extension cc combined with knee flexion cc; 60-second interval; 3-repetition knee extension ecc combined with knee flexion ecc; 60-second interval; 5-repetition knee extension ecc combined with knee flexion ecc. Data collection was obtained using a sampling rate of 100Hz and it was subsequently analyzed with the software *Acqknowledge*, version 4.1 (Biopac Systems, Inc., Goleta, CA, USA). Each individual curve was inspected in order to consider true isokinetic torques within 95% confidence interval of the angular velocity of 60°·s^-1^. The angle of the PT and PT value of the best from five repetitions were retained for analysis (best curve performed by KE and KF in both the cc and ecc actions: KEcc, KEecc, KFcc, KFecc). Composite ratios were derived as follows:

$$Conventional\;ratio=\frac{PT\_KFcc}{PT\_KEcc}$$ (1)

$$Functional\;extension\;ratio=\frac{PT\_KFecc}{PT\_KEcc}$$ (2)

$$Functional\;flexion\;ratio=\frac{PT\_KFcc}{PT\_KEecc}$$ (3)

The above-presented ratios were independent of knee-joint angle. Therefore, the measurements of each muscle group were made at different angles. By using an angular velocity of 60°·s^-1^ and for a range of motion of 85º, the sampling rate of 100 Hz allowed data collection at specific angles. The torque (T) of the KFcc action and the T of the same KFecc action at the angle of the PT of the KEcc action were determined, respectively: T_KFcc at angle PT_KEcc and T_KFecc at angle PT_KEcc. Finally, the T of the KFcc action at the angle of the PT of the KEecc action was also determined (T_KFcc at angle PT_KEecc). T and PT values were expressed in Newton-meter (N·m). The following ratios were also possible:

$$Angle-specific\;conventional\;ratio=\frac{T\_KFcc\;at\;angle\;PT\_KEcc}{PT\_KEcc}$$ (4)

$$Angle-specific\;functional\;extension\;ratio=\frac{T\_KFecc\;at\;angle\;PT\_KEcc}{PT\_KEcc}$$ (5)

$$Angle-specific\;functional\;flexion\;ratio=\frac{T\_KFcc\;at\;angle\;PT\_KEecc}{PT\_KEecc}$$ (6)

### Statistical analysis

Descriptive statistics (minimum, maximum, mean value, standard error of the mean, 95% confidence interval of the mean, and standard deviation) were calculated for chronological age, training experience and anthropometry. In addition, Kolmogorov-Smirnov test was used to check for normality. Comparisons between type of sports were performed using *t-*test analysis. Means and standard deviations of PT, torque at specific angles, conventional and functional ratios were presented for each time-moment and intra-individual mean differences were examined using *t-*test analysis. Effect size was given by d-values of Cohen, which were interpreted as follows: <0.20 (trivial), 0.20 to 0.59 (small), 0.60 to 1.19 (moderate), 1.20 to 1.99 (large), 2.0 to 3.9 (very large), and ≥4.0 (extremely large) [21]. Technical error of measurement (TEM), coefficients of variation (%CV) and intra-class correlation coefficient (ICC) were determined. ICC higher than 0.90 and CV lower than 10% are analytical goals commonly employed in sport and exercise science [22], although recommendations regarding CV in time trial protocols considers a more conservative percentage (<5%) [23]. Additionally, the limits of agreement between ratios were examined by plotting the differences between time-moments relative to mean values of both assessments [24]. Statistical significance was set at *p* < 0.05 and all analyses were performed using the Statistical Package for the Social Sciences version 23.0 (SPSS Inc., IBM Company, Armonk, NY, USA) and GraphPad Prism software (GraphPad Software, Inc., La Jolla, CA, USA).

## Results

Characteristics of the total sample are summarized in Table 1. All variables fit the assumption of normal distribution. Table 2 summarizes descriptive statistics by type of sports [i.e., team sports (soccer, volleyball and roller hockey) and individual sports (combat, swimming, rowing, track and field, tennis and cycling)] and includes results of comparisons between groups. Athletes of contrasting sports did not differ significantly in isokinetic parameters under analysis.

**Table 1:** Descriptive statistics for the total sample (n = 26) and test for normality assumption.

Variable	Unit	Range		Mean			Standard deviation	(Kolmogorov-Smirnov)	
		minimum	maximum	value	SEM	(95% CI)		value	*p*
Chronological age	years	18.6	33.9	24.0	0.7	(22.6 to 25.5)	3.8	0.146	0.16
Training experience	years	2.0	25.0	13.5	1.1	(11.5 to 15.5)	5.6	0.118	0.20
Body mass	kg	58.4	89.2	74.2	1.6	(70.8 to 77.3)	8.4	0.110	0.20
Stature	cm	167.7	193.0	178.3	1.5	(175.1 to 181.1)	7.8	0.157	0.10
Estimated leg length	cm	76.5	94.6	85.0	1.1	(82.7 to 87.0)	5.4	0.088	0.20
Fat mass	%	7.3	30.3	15.3	1.2	(13.1 to 17.3)	6.2	0.135	0.20
Fat mass	kg	4.5	25.6	11.6	1.1	(9.4 to 13.8)	5.7	0.168	0.06
Fat-free mass	kg	51.4	70.5	61.9	1.1	(59.7 to 64.0)	5.6	0.117	0.20
Lower limb lean soft tissue	kg	8.5	11.9	10.4	0.2	(10.1 to 10.8)	0.9	0.152	0.12

Abbreviations: SEM, standard error of the mean; 95% CI, 95% confidence intervals.

**Table 2:** Descriptive statistics (mean ± standard deviation) by type of sports and comparisons between groups.

Parameter	Time-moment	Muscle group	Action	Unit	Type of sports		Mean differences		Student t-test	
					Team sports (n = 14)	Individual sports (n = 12)	Value	(95% CI)	*t*	*p*
PT	M1	KE	cc	N·m	235.3 ± 41.5	212.4 ± 46.1	22.9	(–12.5 to 58.3)	1.334	0.40
PT	M2	KE	cc	N·m	232.7 ± 40.8	209.4 ± 43.7	23.3	(–11.0 to 57.5)	1.403	0.51
PT	M1	KF	ecc	N·m	268.8 ± 68.0	255.7 ± 61.1	13.1	(–39.6 to 65.8)	0.512	0.93
PT	M2	KF	ecc	N·m	270.3 ± 68.2	254.8 ± 60.4	15.5	(–37.0 to 68.1)	0.610	0.99
PT	M1	KF	cc	N·m	138.6 ± 23.9	125.8 ± 27.6	12.9	(–8.0 to 33.7)	1.274	0.54
PT	M2	KF	cc	N·m	136.8 ± 21.7	120.8 ± 27.9	16.0	(–4.0 to 36.1)	1.650	0.47
PT	M1	KE	ecc	N·m	161.7 ± 29.4	143.5 ± 30.9	18.2	(–6.3 to 42.6)	1.534	0.76
PT	M2	KE	ecc	N·m	163.5 ± 24.4	145.3 ± 29.0	18.2	(–3.4 to 39.9)	1.741	0.68
T at angle of PT_KEcc	M1	KF	cc	N·m	115.3 ± 18.9	102.8 ± 21.8	12.5	(–4.0 to 28.9)	1.559	0.28
T at angle of PT_KEcc	M2	KF	cc	N·m	112.8 ± 17.1	100.4 ± 20.8	12.5	(–2.9 to 27.8)	1.674	0.47
T at angle of PT_KEcc	M1	KF	ecc	N·m	120.5 ± 15.5	107.0 ± 18.5	13.5	(–0.2 to 27.3)	2.034	0.26
T at angle of PT_KEcc	M2	KF	ecc	N·m	121.0 ± 13.6	109.0 ± 19.9	12.0	(–1.6 to 25.6)	1.814	0.16
T at angle of PT_KEecc	M1	KF	cc	N·m	116.8 ± 22.0	103.7 ± 29.6	13.0	(–7.8 to 34.0)	1.291	0.36
T at angle of PT_KEecc	M2	KF	cc	N·m	116.4 ± 20.8	100.2 ± 26.0	16.1	(–2.8 to 35.1)	1.757	0.53

Abbreviations: 95% CI, 95% confidence intervals; PT, peak torque; T, torque; KE, knee extensors; KF, knee flexors; cc, concentric action; ecc, eccentric action; M1, time-moment 1; M2, time-moment 2.

Descriptive statistics for time-moment 1 and time-moment 2 are presented in Table 3, which also included intra-individual differences and results of the paired t-tests for all functional parameters: simple and combined variables. For the PT values, significant intra-individual differences were noted in the cc action of the KF (t = 2.062, p≤0.05). However, the difference was interpreted as trivial. The inspection of torque values at specific angles noted a trivial but significant intra-individual difference for the cc action of the KF at the angle of the PT cc action of the KE (t = 2.027, p≤0.05). With one exception differences are not significant for ratios. The mean values of intra-individual differences were significant for the functional extension ratio at the angle of the PT cc action of the KE (t = 2.586, p≤0.05, trivial).

**Table 3:** Descriptive statistics (mean ± standard deviation) by time-moment and intra-individual differences.

Parameter	Muscle group	Action	Unit	Time-moment		Mean difference		Paired *t*-test		Magnitude effect	
				M1 (n = 26)	M2 (n = 26)	Value	(95% CI)	*t*	*p*	*d*	Qualitative
(a) PT	KE	cc	N·m	224.7±44.3	222.0±43.0	2.7	(–0.1 to 5.6)	2.001	0.06	0.06	trivial
(b) PT	KE	ecc	N·m	262.7±64.0	263.1±63.9	–0.4	(–5.1 to 4.3)	0.176	0.86	0.01	trivial
(c) PT	KF	cc	N·m	132.7±26.0	129.4±25.5	3.3	(0.1 to 6.6)	2.062	≤0.05	0.13	trivial
(d) PT	KF	ecc	N·m	153.3±30.9	155.1±27.7	–1.8	(–6.4 to 2.8)	0.797	0.43	0.06	trivial
(e) T at angle of PT_KEcc	KF	cc	N·m	109.5±20.9	107.1±19.6	2.5	(–0.1 to 5.0)	2.027	≤0.05	0.12	trivial
(f) T at angle of PT_KEcc	KF	ecc	N·m	114.3±18.0	115.5±17.5	–1.2	(–3.9 to 1.5)	0.920	0.37	0.07	trivial
(g) T at angle of PT_KEecc	KF	cc	N·m	110.8±26.1	108.9±24.3	1.8	(–0.9 to 4.6)	1.367	0.18	0.08	trivial
Conventional ratio	(c)/(a)			0.60±0.08	0.59±0.08	0.01	(–0.01 to 0.02)	1.026	0.32	0.13	trivial
Conventional ratio at angle of PT_KEcc	(e)/(a)			0.49±0.07	0.49±0.08	0.01	(–0.01 to 0.01)	1.420	0.17	0.01	trivial
Functional extension ratio	(d)/(a)			0.69±0.11	0.71±0.10	–0.02	(–0.04 to 0.01)	1.911	0.07	0.19	trivial
Functional extension ratio at angle of PT_KEcc	(f)/(a)			0.52±0.09	0.53±0.09	0.01	(–0.01 to 0.02)	2.586	≤0.05	0.11	trivial
Functional flexion ratio	(c)/(b)			0.53±0.17	0.52±0.16	0.01	(–0.01 to 0.03)	1.845	0.08	0.06	trivial
Functional flexion ratio at angle of PT_KEecc	(g)/(b)			0.83±0.10	0.84±0.08	–0.01	(–0.02 to 0.01)	1.032	0.31	0.11	trivial

Abbreviations: M1, time-moment 1; M2, time-moment 2; 95% CI, 95% confidence interval; PT, peak torque; T, torque; KE; knee extensors; KF; knee flexors; cc, concentric; ecc, eccentric.

**Fig 1:**
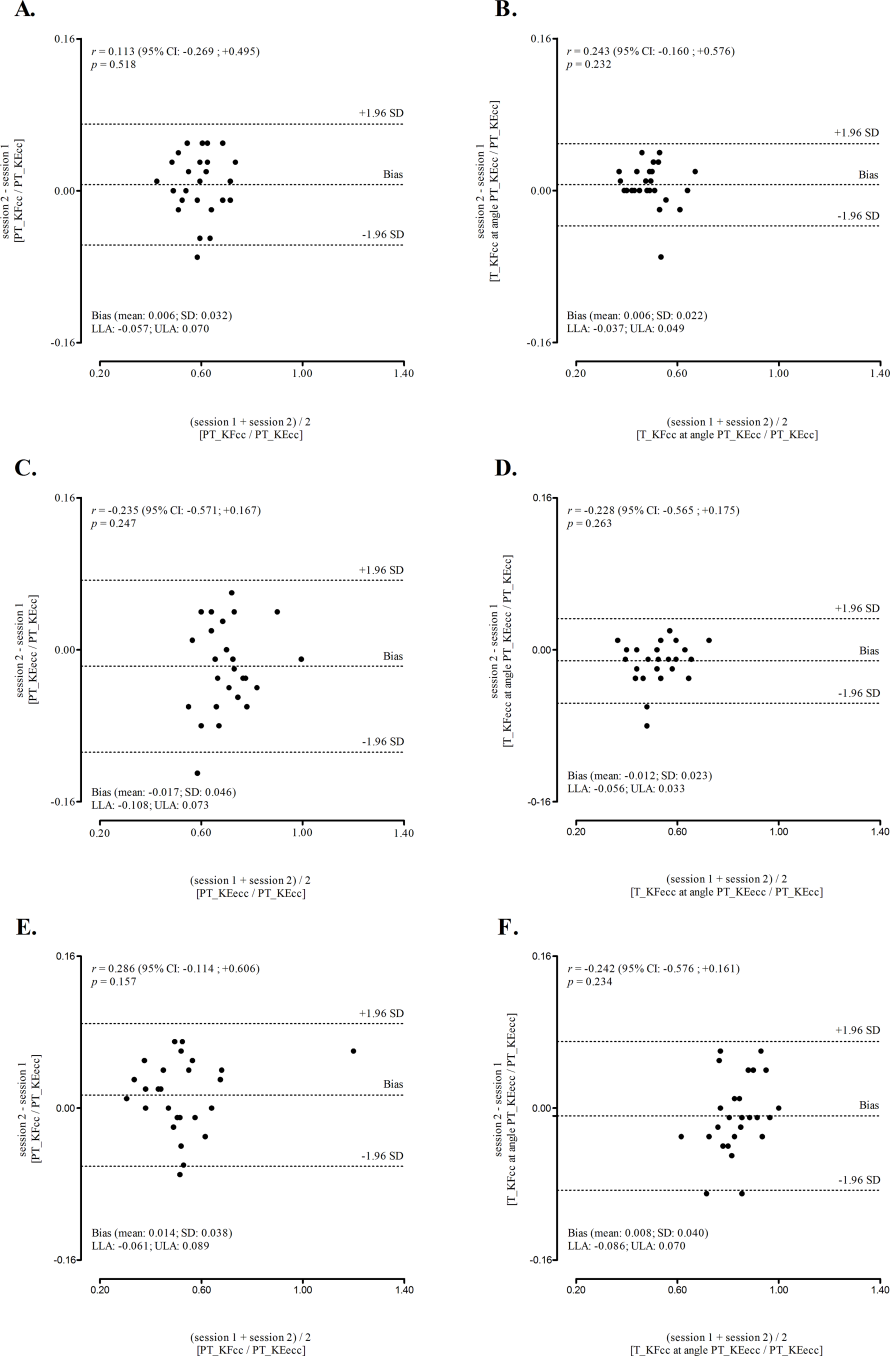
**Agreement of repeated measures for isokinetic ratios: conventional ratio (PT_KFcc/PT_KEcc; Panel A), angle-specific conventional ratio (T_KFcc at angle PT_KEcc/PT_KEcc; Panel B), functional extension ratio (PT_KFecc/PT_KEcc; Panel C), angle-specific functional extension ratio (T_KFecc at angle PT_KEcc/PT_KEcc; Panel D), functional flexion ratio (PT_KFcc/PT_KEecc; Panel E), and angle-specific functional flexion ratio (T_KFcc at angle PT_KEecc/PT_KEecc; Panel F).** The relation between residuals (absolute mean differences between session 2 and session 1), the corresponding mean (heteroscedasticity diagnostic), and the 95% confidence intervals (95% CI) are also presented. The dashed lines represent 95% limits of agreement (±1.96 SD); lower limits of agreement (LLA) and upper limits of agreement (ULA).

Table 4 reports the TEM and associated CV, which range between 2.33%-5.19% for PT value and 4.18%-4.46% for torque values at specific angles. Regarding composite variables, with one exception (functional flexion ratio from PTs obtained in different angles), all %CV were lower than 5% (3.36%-4.29%). The %CV for the functional ratio between PT of the cc action of the KF divided by the PT of the ecc action of the KE was 5.71%. For all measurements (simple and ratios) the ICC values were always over 0.95. Finally, Fig 1 illustrates the discrepancies of repeated measurement (Y-axes: session 2 minus session 1) for conventional ratio (panels A and B), for functional extension ratio (panels C and D) and for functional flexion ratios (panels E and F). The panels don’t suggest heterocedasticity among axes (with X-axes being the mean of the repeated measurement) neither by visual inspection of the graphic nor based on statistics.

**Table 4:** Technical error of measurements, coefficient of variation and intra-class correlation for the simple and combined variables.

Parameter	Muscle group	Action	Unit	TEM	%CV	ICC	
						Coefficient	(95% CI)
(a) PT	KE	cc	N·m	5.2	2.33	0.993	(0.983 to 0.997)
(b) PT	KE	ecc	N·m	8.1	3.08	0.992	(0.981 to 0.996)
(c) PT	KF	cc	N·m	6.2	4.73	0.974	(0.942 to 0.988)
(d) PT	KF	ecc	N·m	8.0	5.19	0.961	(0.914 to 0.983)
(e) T at angle of PT_KEcc	KF	cc	N·m	4.6	4.25	0.976	(0.947 to 0.989)
(f) T at angle of PT_KEcc	KF	ecc	N·m	4.8	4.18	0.962	(0.916 to 0.983)
(g) T at angle of PT_KEecc	KF	cc	N·m	4.9	4.46	0.981	(0.958 to 0.992)
Conventional ratio	(c)/(a)			0.02	3.36	0.956	(0.902 to 0.980)
Conventional ratio at angle of PT_KEcc	(e)/(a)			0.02	4.08	0.978	(0.951 to 0.990)
Functional extension ratio	(d)/(a)			0.03	4.29	0.950	(0.889 to 0.978)
Functional extension ratio at angle of PT_KEcc	(f)/(a)			0.02	3.81	0.983	(0.962 to 0.992)
Functional flexion ratio	(c)/(b)			0.03	5.71	0.987	(0.971 to 0.994)
Functional flexion ratio at angle of PT_KEecc	(g)/(b)			0.03	3.59	0.948	(0.885 to 0.977)

Abbreviations: PT, peak torque; T, torque; KE, knee extensors; cc, concentric; ecc, eccentric; TEM, technical error of measurement; CV, coefficient of variation; ICC, intra-class correlation; 95% CI, 95% confidence interval.

## Discussion

The present research examined the reliability of traditional and new isokinetic simple and combined indicators, resulting from KE and KF measurements in maximal cc and ecc tests performed at 60°·s^-1^. The main results showed suitable levels of systematic bias, absolute and relative reliability for PT, torque at specific angles, conventional and functional ratios, and conventional and functional ratios at specific angles, recorded from 26 adult male athletes who were tested on two separate occasions, with an interval of one week. Allowing for sample size, type of sports did not seem to affect the parameters under analysis.

The presence of systematic bias was assessed using the paired t-test and the magnitude effect. The paired t-test was able to detect statistically significant differences only in three of the thirteen studied isokinetic indicators: PT of KF in the cc test (lower on retest), torque of KF at angle of PT of KE in the cc test (lower on retest), and functional extension ratio at angle of PT of KE in the cc test (higher on retest, due to a non-significant increased ecc angle-specific flexor torque and a non-significant decreased cc extensor PT). Learning effects (better on retest) or fatigue effects (worse on retest) are the most probable explanations for systematic bias [22]. However, a one-week interval between test sessions appears to be sufficient to recover from the previous test, although these differences were primarily for the flexor muscles, which are usually less accustomed to producing maximal levels of muscle strength. Nevertheless, ten of the thirteen isokinetic indicators did not significantly differ between repeated time-moments and all magnitude effects were interpreted as trivial. Overall, the amount of systematic bias was acceptable. Lund et al. [25] tested thirteen healthy participants (9 women, 5 men; mean age: 32 years; range: 18–55 years) in 5 time-moments using a Biodex System 3 dynamometer and found no systematic effect over time for knee extension and flexion performed at 60°·s^-1^.

The absolute reliability was verified using TEM and (%)CV. Low TEM and CV below the proposed analytical goal of 5%, with the exception of the ecc knee flexion PT (5.2%) and functional flexion ratio (5.7%), suggested relatively little within-subject variation. Nevertheless, it is likely that measurement errors between sessions might be more related to biological or mechanical variation (i.e. random error) [22]. In a study by Birmingham et al. [26], a CV cut-off point of 8% resulted in correctly identification of 95% of maximal efforts.

The relative reliability was determined using the ICC. High ICCs for all the isokinetic indicators confirmed that the stability of the measurements over time was good. In general, the subjects maintained their position in the sample following the retest. Gleeson and Mercer [27] suggested that CV < 6.1% and ICCs ≥ 0.88 can be interpreted as suitable reliability in isokinetic strength testing. Lund et al. [25] reported ICCs ≥ 0.89 for KEcc and KFcc PT across 5 time-moments. Feiring et al. [28] also reported high ICCs for the KEcc (0.95) and KFcc (0.98) PT using a 7-days interval between testing sessions.

There is a lack of studies concerning the reliability of other isokinetic indicators, beyond the PT. Additionally, there are few studies that present results for KE and KF in cc and ecc actions using Biodex isokinetic dynamometers. Carvalho et al. [29] presented higher CV and lower ICCs for KEcc (%CV = 4.9–8.1; ICC = 0.89–0.95), KFcc (%CV = 3.9–16.5; ICC = 0.78–0.99), KEecc (%CV = 6.0–15.1; ICC = 0.74–0.95) and KFecc (%CV = 5.3–17.7; ICC = 0.72–0.97) using a closely related methodology of isokinetic testing in adolescent basketball players. At 60°·s^-1^, Ayala et al. [30] also presented higher CV and ICCs for KEcc (%CV = 16.45; ICC = 0.71), KFcc (%CV = 13.33; ICC = 0.78), KEecc (%CV = 17.09; ICC = 0.81) and KFecc (%CV = 8.99; ICC = 0.90) adopting a prone position.

The present study has several limitations that should be mentioned. The sample size was small, based on convenience and male subjects; therefore, the results are not generalizable. Further research in other populations is justified. Only two test sessions were considered; a third test session with an interval of one week could be more informative about the effect of familiarization between test sessions. Only the dominant lower limb was tested; further testing is necessary to assess the bilateral differences. Only tests performed at 60°·s^-1^ were used; further testing is necessary using other angular velocities, especially higher angular velocities when isokinetic dynamometers with higher sampling rates are available. The validity, responsiveness and clinical significance (especially relevant for subjects with knee injury) of the isokinetic indicators were not tested, issues that are lacking in the literature.

This information has practical implications in the management of research projects. Standardization of testing procedures is highly desirable, especially in terms of equipment calibration, warm-up procedures, familiarization training, number of submaximal trial repetitions, subject positioning and stabilization, axis alignment, testing setups, anatomical reference angle determination, verbal and/or visual feedback, gravity correction and testing protocol definition. Standardization of data collection, processing and analysis procedures are equally highly recommended.

## Conclusions

In summary, results evidenced that the traditional and new isokinetic simple and combined indicators considered in this study are reliable to assess muscle strength and function in adult male athletes when derived from an isokinetic dynamometer that provides sampling rates of at least 100Hz, using a closely related methodology. Isokinetic indicators obtained during a single testing session seem to be sufficiently reliable. This can be especially important when it is not possible to bring the athletes to the laboratory prior to the test session for familiarization purposes. In fact, isokinetic dynamometry is considered the gold standard for the objective measurement of dynamic muscle strength and function. Strategies to minimize measurement error are of extreme relevance. New and ground-breaking concepts and methods for reporting reliable isokinetic data must also be explored, for example, considering angle-specific information in a more specific and meaningful manner.

## Supporting information

S1 File Full dataset. (XLSX)
